# Genome Sequence of a Novel Reassortant H3N2 Avian Influenza Virus from Domestic Mallard Ducks in Eastern China

**DOI:** 10.1128/genomeA.00221-12

**Published:** 2013-04-11

**Authors:** Qunhui Li, Qingqing Zhao, Min Gu, Jie Zhu, Xiaobing Gu, Guo Zhao, Qingtao Liu, Xiaoquan Wang, Xiaowen Liu, Xiufan Liu

**Affiliations:** Animal Infectious Disease Laboratory, College of Veterinary Medicine, Yangzhou University, Yangzhou, Jiangsu, Chinaa;; Ministry of Education Key Lab for Avian Preventive Medicine, Yangzhou University, Yangzhou, Jiangsu, Chinab

## Abstract

The H3 subtype avian influenza virus (AIV) can provide genes for human influenza virus through gene reassortment, which raises great concerns in terms of its potential threat to human health. Here, we report the complete genome sequence of a novel H3N2 AIV isolated from domestic ducks in the Jiangsu province of eastern China in 2004, which is a natural recombinant virus whose genes are derived from H3N8, H5N1, H5N2, H11N2, H4N6, and H1N1 AIVs. This genome will help to understand the epidemiology and molecular characteristics of H3N2 influenza virus in eastern China.

## GENOME ANNOUNCEMENT

Avian influenza (AI) is caused by type A influenza viruses, which mainly result in systemic or respiratory diseases in birds. All known influenza A virus subtypes differ with respect to two surface glycoproteins, hemagglutinin (HA) (H1 to H17) and neuraminidase (NA) (N1 to N10) (1, 2). It is notable that the results of a recent epidemiological investigation of different-HA-subtype avian influenza viruses (AIVs) showed that the H3 subtype is the predominant subtype among low-pathogenic AIVs, and the seasonal variations in isolates of the H3 subtype AIVs are consistent with those of human H3 subtype influenza viruses (3). Some research predicts that H3 subtype AIVs will be capable of directly infecting humans through gene reassortment (4, 5). Previous studies demonstrated that the Hong Kong pandemic influenza virus (H3N2) in 1968 was a reassortant with avian (H3) PB1 and HA genes and six other genes from the human (H2N2) virus (6). Therefore, these findings emphasize the importance of H3 AIV surveillance for understanding the genesis and emergence of novel reassortants with pandemic potential.

In this study, strain A/duck/Jiangsu/26/2004 (H3N2) was isolated from apparently healthy domestic mallard ducks in the Jiangsu province of eastern China. PCR was performed by using the universal primers for influenza A virus (7). These PCR products were purified and sequenced on an ABI 3730 capillary DNA-sequencing instrument. Genome sequences were aligned by using ClustalW in the Molecular Evolutionary Genetics Analysis (MEGA) 5.0 software.

The complete genome of the strain consists of eight segments of negative-sense single-stranded RNA molecules, including polymerase basic 2 (PB2), PB1, polymerase acidic (PA), HA, nucleoprotein (NP), NA, matrix (M), and nonstructural (NS) proteins. The full lengths of these segments are 2,341, 2,341, 2,233, 1,765, 1,565, 1,467, 1,027, and 890 nucleotides, respectively. The strain possessed the low-pathogenic influenza A virus sequence PEKQTR↓G at the cleavage site. Analysis of potential glycosylation sites of the isolate revealed that there were 6 potential *N*-linked glycosylation sites in HA (positions 8, 22, 38, 165, 285, and 483), while there were 7 in NA (positions 61, 69, 70, 146, 200, 234, and 402). Furthermore, there were no changes in the length of the NA stalk region and the NS1 protein.

Sequence analysis showed that the HA gene showed the highest sequence homology (96%) with that of the isolate A/duck/Siberia/100/2001 (H3N8). PB1 and NP showed the highest sequence homologies (99%) with those of the virus strains A/R (duck/Mongolia/54/01-duck/Mongolia/47/01) (H5N1) and A/water/Xinjiang/2009 (H5N1). PB2 and PA showed the highest sequence homologies (99%) with those of the isolates A/aquatic/Korea/MA81K/2007 (H5N2) and A/duck/Jiangxi/1286/2005 (H5N2), respectively. The NA gene showed the highest sequence homology (99%) with that of the isolate A/swan/Shimane/48/1997 (H11N2). The M gene showed the highest sequence homology (99%) with that of the isolate A/wild bird/Korea/YS109/2007 (H4N6). In addition, the NS gene was most closely related to that of the isolate A/mallard/Bavaria/185-8/2008 (H1N1). Thus, we isolated a novel multiple-gene reassortant H3N2 influenza virus whose genes were derived from H3N8, H5N1, H5N2, H11N2, H4N6, and H1N1 AIVs.

Therefore, the genome information of A/duck/Jiangsu/26/2004 (H3N2) will help in the investigation of the epidemiology of AIV in domestic ducks in China.

### Nucleotide sequence accession numbers. 

The complete genomic sequence of A/duck/Jiangsu/26/2004 (H3N2) was deposited in GenBank under the accession no. KC261666 to KC261673.
